# Supplementary material for: Transmissible Gastroenteritis Virus Binding to Red Blood Cells Disrupts Iron Homeostasis and Promotes Viral Infection
Source: Vet Sci. 2026 Jan 3;13(1):42. doi: 10.3390/vetsci13010042 (PMC12846369; doi:10.3390/vetsci13010042)

Transmissible Gastroenteritis Virus Binding to Red Blood Cells Disrupts Iron Homeostasis and Promotes Viral Infection

Figure S1. Original plaque assay for the role of SA in TGEV binding to RBCs

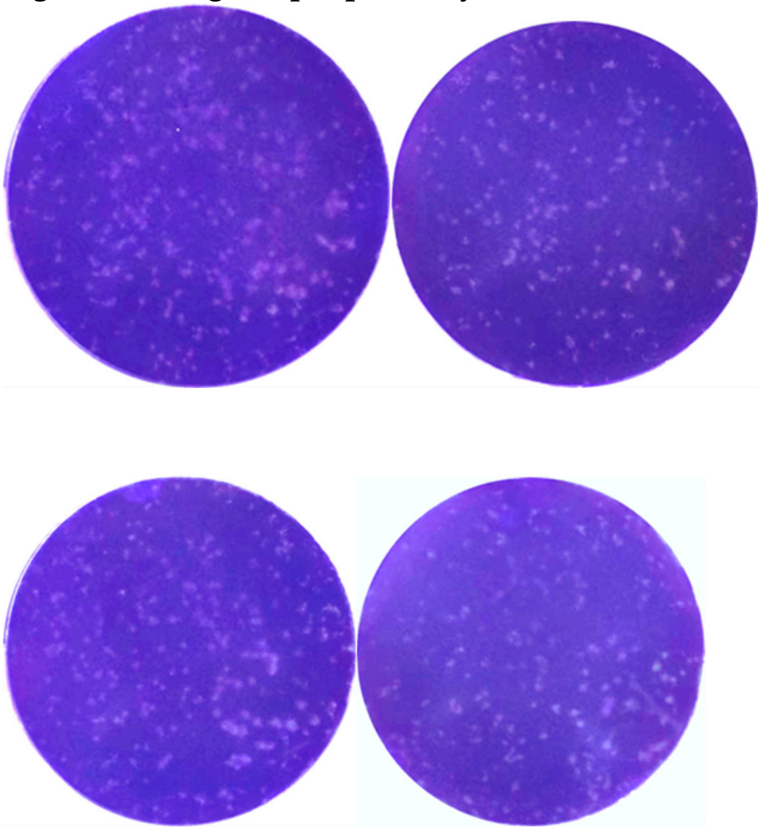

Figure S2. Original image depicting the effects of DFOM or FAC on TGEV infection.

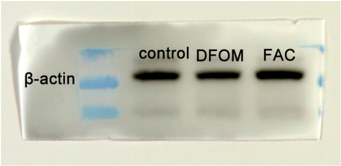

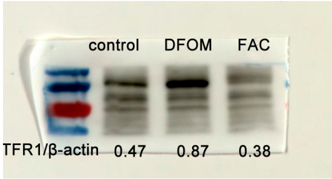

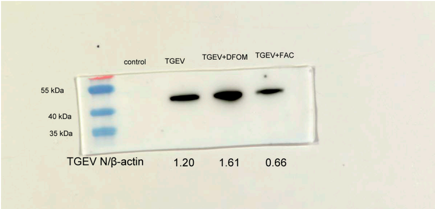

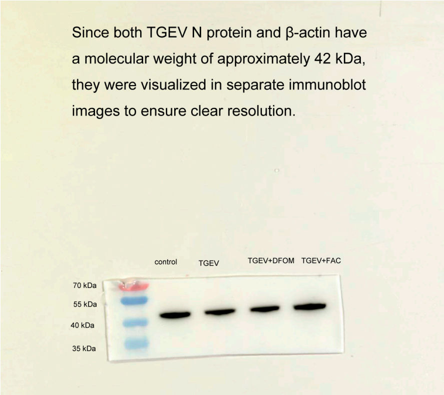

Supplement: Supplementary file 1 [file vetsci-13-00042-s001.zip › vetsci-3992115-supplementary.pdf]
